# The association between BMI development among young children and (un)healthy food choices in response to food advertisements: a longitudinal study

**DOI:** 10.1186/s12966-016-0340-7

**Published:** 2016-02-09

**Authors:** Frans Folkvord, Doeschka J. Anschütz, Moniek Buijzen

**Affiliations:** Behavioural Science Institute, Radboud University, Thomas van Aquinostraat 8.01.03, 6526 GD Nijmegen, Netherlands; From the Applied Social Science and Behavioral Economics Research Group, Universitat Oberta de Catalunya, Barcelona, Spain

**Keywords:** BMI, Childhood obesity, Food advertisements, Long-term effects, Snack intake

## Abstract

**Background:**

Previous studies have focused on the acute effects of food advertisements on the caloric intake of children; however, the long-term effects of this food cue reactivity on weight gain have not been examined. The main aim of this study was to explore if reactivity to food cues in an advertisement was associated with weight status two years later.

**Methods:**

Children wo had previously taken part in an experiment investigating the impact of advergames on food intake had their height and weight re-measured two years later, for assessment of body mass index (BMI). A within-subject design was used to test the associations between food choices and BMI over time. In the previous experiment, children played an advergame that promoted energy-dense snacks, fruit, or nonfood products, or did not play an advergame (control condition). After playing the game, the free intake of energy-dense snacks and fruits was measured.

**Results:**

Children who ate more apple after playing an advergame promoting energy-dense snacks had a lower BMI two years later. Consumption of energy-dense snacks after playing an advergame promoting energy-dense snacks was not associated with BMI two years later. In other condition, no association was found between food intake and BMI after two years .

**Conclusions:**

The findings suggest that coping with environmental cues that trigger unhealthy eating behavior is associated with the body mass index of young children two years later. This might imply that learning to respond to food cues by choosing healthy options might prevent children from excessive weight gain.

This trial was registered at as ISRCTN17013832.

**Electronic supplementary material:**

The online version of this article (doi:10.1186/s12966-016-0340-7) contains supplementary material, which is available to authorized users.

## Background

Childhood obesity has more than doubled in children over the last three decades [1]. In parallel with this, total expenditure for food advertisements to children and the availability of these foods has increased enormously [2]. Many empirical studies and reviews have concluded that the intense advertising of energy-dense, micronutrient-poor food and beverages--which is designed to stimulate consumption--is a probable causal factor in childhood obesity [3–5].

In particular exposure to food-promoting ‘advergames’ has been shown to have strong effects on eating behavior of children [6–9]. Advergames request active attention and participation, and the food cues are highly embedded in the content of the game, thereby reducing the cognitive elaboration of the marketing message among children [9–14]. Considering that these attractive food advertisements are mostly promoting energy-dense snacks, high in salt, sugar and fat, and have low nutritional value [5], it becomes difficult for children to inhibit their responses towards energy-dense food, and choose a healthier option if they crave for the advertised food.

The cue reactivity theory predicts that food cues in advergames signal food intake, that subsequently act as conditioned stimuli that trigger conditioned responses, such as craving and actual eating behavior [15]. Food advertisements capture attention and trigger neurological reward systems [16, 17], which induces craving and motivating behavior toward palatable and energy-dense foods [17]. A heightened responsiveness to external cues that predict food intake has been identified by the incentive-sensitization model of obesity as an important mechanism that stimulates overeating [18] and weight gain in some individuals [19, 20]. The opposite process might also hold, in which a lower responsiveness to external cues or a better inhibition system leads to less weight gain.

Until now, no study has examined if the reactivity to food cues in advertisements is associated with future weight gain. This is remarkable, because this reactivity is generally considered to be the most important environmental instigator of the obesity epidemic [21, 22]. For example, a review showed that the consumption of energy-dense food with a high glycaemic load contributed to the rates of overweight and obesity in the US [23]. In addition, Yokum et al. [24] demonstrated that adolescents who showed greater activity in brain areas related to reward regions during commcerials for energy-dense foods, had gained more weight in a follow-up. When external food cues trigger craving for food, it might be that children who choose to eat energy-dense food to fulfill their craving, gain more weight than children who choose to eat a more healthy option, such as fruit.

The main objective of this study is to explore if children’s reactivity to food cues in an advertisement at time point one (T1) is related to their body mass index after a period of two years (T2). It is expected that the amount of energy-dense snacks consumed at T1 will be positively related to BMI at T2, in particular after playing an advergame promoting energy-dense snacks, because this game specifically induces craving for energy-dense snacks (H1). Furthermore, it is expected that the amount of fruit that children consumed at T1 will be negatively related with BMI at T2, in particular after playing an advergame promoting energy-dense snacks (H2).

## Methods

### Experimental design and stimulus materials

In a previous study (T1), children were randomly assigned to 1 of 4 conditions, which involved playing (1) an energy-dense snacks advergame, (2) a fruit advergame (3) a nonfood advergame or (4) no game at all. After playing the advergame, children were seated at a table and four different pre-weighed bowls were placed in front of them, from which they could eat *ad libitum*. Two bowls contained energy-dense food snacks: (1) jelly candy (cola bottles) and (2) milk chocolate candy shells, and two bowls contained sliced fruit pieces: (3) banana and (4) apple. Food choices at T1 was considered as cue reactivity for the present study. After eating, BMI was assessed (for more information about this study see [6]). Two years later (T2), the same children were approached whereby BMI was measured again. Within game conditions it was examined whether the amount of food eaten after playing the game (or control condition) was associated with BMI at T2.

### Participants

Of the 270 participants included in the previous study [6], 218 participants were weighed and measured at T2 (mean age = 11.13 y; SD = 0.76 y; grades 5–6; mean BMI = 18.31; SD = 2.73, BMI range =13.65–30.83) from six different primary schools in the Netherlands; 51.4 % of the participants were boys. Using international cut-off scores [24], among the children in this follow up study (T2), 12 children were underweight, 170 children were normal weight, 33 children were overweight, and 3 children were obese.

### Measures

#### Caloric intake

To measure caloric intake after playing the advergame at T1, we allowed the children to eat for 5 min, ad libitum. We weighed the amount of snack food that a child ate before each child entered the room and weighed again after eating. We used a professional balance scale to estimate to the nearest 0.1 gram. We recalculated the number of grams that a child ate of the jelly candy, milk-chocolate candy shells, banana, and apple, in kilocalories for use as independent measures in the current study. The amount of energy-dense snack food that a child ate was the sum of the kilocalorie intake of jelly candy and milk chocolate candy shells, and the amount of fruits was the sum of the kilocalorie intake of banana and apple.

#### BMI

BMI was measured at T1 and at T2. BMI was measured as weight (kg)/height^2^ (cm). Weight was measured to the nearest 0.1 kg while the children were wearing clothing but no shoes. Height was also measured according to standard procedures to the nearest 0.5 cm.

### Procedure

The Ethics Committee Faculty of Social Sciences at the Radboud University approved the current study. The study was conducted between February and June 2014. After obtaining consent from the schools to participate, a letter with detailed information regarding the study and the possibility to withdraw from participation was sent to the parents of the children. Both the parents and the children were given the opportunity to decline participation. To both parents and children it was explained that we would measure height and weight, that all information would be confidential, and that children could stop participation at any moment. Around 82 % (*N* = 270) of the children who participated at T1 also participated at T2 (*N* = 218).

At T2 children were individually tested with the same equipment at their schools during regular school hours. The teacher assigned the children (in alphabetical order) to the experimenter and the experimenter brought each child to a separate room where there weight and height were measured. The children were then accompanied back to their classrooms, and the experimenter invited the next child to participate.

### Statistical analyses

The means and SDs for all variables are presented separately for each condition in Table 1. To test the hypotheses, two separate sets of linear regression analyses^1^ were conducted, with intake of jelly candy, milk-chocolate candy shells, banana, and apple as independent variables. Because BMI at T1 was highly correlated (*r =* 0.93) with BMI at T2, two separate sets of analyses were conducted, one with and without BMI at T1 included as a factor in the models. Each set of linear regression analyses contained four regression analyses for each condition seperately. The results are shown in Table 2. The two-sided adjusted *P* value that was considered significant was 0.05.

**Table 1:** Variables measured by the condition^ab^

	Energy-dense snack advergame (*n =* 57)	Fruit advergame (*n =* 54)	Nonfood advergame (*n =* 51)	Control advergame (*n =* 56)
Sex (= boy)	40.4 %	59.3 %	51 %	55.4 %
Age (y)	8.9 ± 0.7	9.0 ± 0.7	8.9 ± 0.8	8.8 ± 0.8
BMI T1	17.9 ± 2.5	17.5 ± 2.9	17.1 ± 1.9	16.7 ± 1.9
BMI T2	18.9 ± 2.8	18.6 ± 3.2	17.9 ± 2.2	17.8 ± 2.5
Jelly candy intake (kcal)	83.0 ± 88.2	78.7 ± 75.2	61.2 ± 49.8	41.7 ± 41.3
Milk chocolate candy shells intake (kcal)	82.1 ± 82.6	74.3 ± 82.2	45.8 ± 63.0	41.1 ± 46.1
Banana intake (kcal)	15.7 ± 18.6	18.8 ± 23.2	15.3 ± 20.2	13.0 ± 17.0
Apple intake (kcal)	17.1 ± 16.1	13.9 ± 14.4	10.4 ± 10.5	11.0 ± 11.7
Total energy-dense food intake (kcal)	165.1 ± 109.6	153.1 ± 118.1	107.0 ± 86.5	82.8 ± 75.0
Total fruit intake (kcal)	32.8 ± 26.0	32.7 ± 29.9	25.7 ± 24.7	24.0 ± 23.1
Total calorie intake (kcal)	197.9 ± 114.3	185.8 ± 119.8	132.7 ± 87.7	106.8 ± 76.7

^a^Mean ± SD (all such values). *VAS* visual analogue scale

^b^ *N* = 218

**Table 2:** Linear regression analyses with BMI T2 as dependent variable^a^

	Energy-dense advergame		Fruit advergame		Nonfood advergame		Control	
	BMI T2	BMI T2	BMI T2	BMI T2	BMI T2	BMI T2	BMI T2	BMI T2
Jelly candy intake (kcal)	-0.04 (0.00)	-0.00 (0.00)	-0.04 (0.00)	-0.01 (0.00)	-0.11 (0.00)	0.00 (0.00)	-0.02 (0.00)	0.00 (0.01)
Milk chocolate candy shells intake (kcal)	0.03 (0.00)	0.00 (0.00)	0.12 (0.00)	0.00 (0.00)	0.06 (0.00)	-0.00 (0.00)	-0.02 (0.00)	0.00 (0.01)
Banana intake (kcal)	-0.05 (0.01)	0.00 (0.01)	0.05 (0.01)	0.01 (0.01)	0.04 (0.01)	0.01 (0.01)	0.11 (0.01)	0.01 (0.02)
Apple intake (kcal)	-0.33^b^ (0.01)	-0.01 (0.01)	0.02 (0.01)	-0.01 (0.01)	-0.03 (0.01)	-0.02 (0.02)	-0.06 (0.01)	-0.02 (0.02)
BMI T1		0.99 (0.06)^b^		1.00 (0.06)^b^		1.02 (0.09)^b^		0.88 (0.13)^b^
*Effect size (R* ^*2*^)	*0.12*	*0.86*	*0.02*	*0.84*	*0.01*	*0.73*	*0.02*	*0.42*

^a^ *N* = 218

*P* < 0.05

^b^ *P* < 0.01

## Results

The results of the first set of linear regression analyses are shown in Table 2. For the children who played the advergame promoting energy-dense snacks, *F*(4, 52) = 1.750, *P =* 0.15, *R*^*2*^ *=* 0.12, no effect was found at BMI T2 for the amount of jelly candy intake (β = 0.03, *P =* 0.79), milk-chocolate candy shells intake (β = - 0 .04, *P =* 0.82), and banana intake (β = -0.05, *P =* 0.68), and a significant effect of apple intake (β = -0.33, *t* (51) = - 2.52, *P =* 0.01). For the children who played the advergame promoting fruit, F (4, 49) = 0.198, *P =* 0.94, *R*^*2*^ *=* 0.02, the advergame promoting nonfood products, F (4, 46) = 0.161, *P =* 0.80, *R*^*2*^ *=* 0.01, and the control condition, F (4, 49) = 0.198, *P =* 0.43, *R*^*2*^ *=* 0.02, no significant effects were found for the food intake at BMI T2.

The results of the second set of linear regression analyses are shown in Table 2. For the children who played the advergame promoting energy-dense snacks, F (5, 55) = 55.442, *P =* 0.00, *R*^*2*^ *=* 0.84, no effect was found at BMI T2 for the amount of jelly candy intake (β = 0.03, *P =* 0.79), milk-chocolate candy shells intake (β = - 0 .04, *P =* 0.82), banana intake (β = -0.05, *P =* 0.68), and apple intake (β = -0.13, *P =* 0.15), and a significant effect for BMI at T1(β = 0.99, *t* [50] = 17.00, *P =* 0.00) . For the children who played the advergame promoting fruit, F (5, 48) = 66.915, *P =* 0.00, *R*^*2*^ *=* 0.86), no effect was found at BMI T2 for the amount of jelly candy intake (β = -0.01, *P =* 0.54), milk-chocolate candy shells intake (β = 0.00, *P =* 0.18), banana intake (β = 0.01, *P =* 0.16), and apple intake (β = -0.01, *P =* 0.63), and a significant effect for BMI at T1(β = 1.00, *t* [48] = 16.49, *P =* 0.00). For the children who played the advergame promoting nonfood products, F (5, 50) = 27.506, *P =* 0.00, *R*^*2*^ *=* 0.73, no effect was found at BMI T2 for the amount of jelly candy intake (β = 0.00, *P =* 0.75), milk-chocolate candy shells intake (β = - 0.01, *P =* 0.62), banana intake (β = 0.08, *P =* 0.34), and apple intake (β = -0.02, *P =* 0.23), and a significant effect for BMI at T1(β = 1.02, *t* [50] = 11.62, *P =* 0.00). For the children who were in the control condition, F (5, 50) = 8.817, *P =* 0.00, *R*^*2*^ *=* 0.42), no effect was found at BMI T2 for the amount of jelly candy intake (β = -0.00, *P =* 0.70), milk-chocolate candy shells intake (β = 0.00, *P =* 0.95), banana intake (β = 0.01, *P =* 0.74), and apple intake (β = -0.02, *P =* 0.33), and a significant effect for BMI at T1(β = 0.88, *t* [50] = 6.53, *P =* 0.00). Scatter plots (see Additional file 1) were plotted to visualize the results with the regression lines superimposed by condition.

## Discussion

The main aim of this study was to explore if reactivity to food cues in an advertisement was associated with weight status two years later. At T1, children played one of three advergames (promoting energy-dense snacks, fruit or nonfood products) or played no game at all and could eat ad libitum after playing, and their BMI was measured. At T2, two years later, BMI of the same children was assessed again. Hypotheses were tested with separate hierarchical regression analyses within conditions.

The results showed that the intake of (both of) the energy-dense snack foods was not related to BMI at T2, in none of the four conditions. These findings refute H1. Furthermore, we found that apple intake was negatively related to BMI at T2, but only for the children who played the energy-dense advergame. For banana intake we found no relationship with the BMI measurements. These findings support H2 partly. The relations between apple intake and BMI at T2 was not present when BMI at T1 was included as a factor. The main reasons for the different findings was that BMI at T1 was strongly positively related with BMI at T2, in all four conditions, and that BMI at T1 was strongly negatively related with apple intake among the children in the energy-dense advergame.

Interestingly, when food cues of energy-dense snacks induced craving for food, those children who chose to fulfill this craving with eating a healthier option--that is, apple--had a lower BMI two years later than the other children. We found this effect only for the children who played the advergame promoting energy-dense snacks, and not for the children in the other conditions. Because BMI at T1 was strongly negatively correlated with apple intake among children who played the energy-dense advergame, it seems that children with a lower BMI at T1 decided to eat the healthier option to satisfy the induced craving. Scatterplots of the results of apple intake by BMI at T1 and at T2 with the regression line superimposed for children who played the energy-dense advergame or in the other conditions illustrate that BMI at T1 and at T2 was strongly negatively related with apple intake among the children who played the advergame promoting energy-dense snacks, and not among the children in the other conditions. Furthermore, scatterplots of candy intake by BMI at T1 and at T2 illustrate that there is no relation between energy-dense intake and BMI at T1 or at T2 for children who played the advergame promoting energy-dense snacks, or for children in the other conditions. These plots support the interpretation that children with a lower BMI at T1 who played the energy-dense advergame satisfied the induced craving by eating apples.

These results suggest that it might be beneficial to stimulate children to consume healthy snacks, instead of energy-dense snacks, when food cues in advertisements induce craving for the advertised food. Apple consumption when craving for food can affect body weight successfully, because apples are high in water and fiber and low in energy-density, and fulfill hunger to a larger extent than energy-dense snacks [16]. In contrast, consuming energy-dense snacks activates brain activity in the reward system and stimulates overeating, instead of fulfilling craving [25–27]. Therefore, it may be a better strategy to teach children to consume healthy food, like fruit and vegetables, rather than teaching them to avoid palatable foods when craving for food has been induced by a food advertisement.

Substituting energy-dense snacks, like candy, for fruits has been shown to be an effective weight-management strategy in short-term clinical studies [28]. Furthermore, in families where parents were encouraged to increase fruit and vegetable intake, significant decreases were shown in the percentage of overweight among both parents and children [29]. Most dietary approaches for obesity prevention attempt to limit intake of energy-dense foods, but this might be perceived as an unwanted restriction for children who find these foods rewarding [29]. Because the feeling that they are restricted can lead to increases in preference for these foods [30], it might be more beneficial to teach children to consume healthy high-nutrient dense foods when they feel craving for food [31].

A possible explanation why we did not find a relationship between apple intake and BMI at T2 when we controlled for BMI at T1, is that apple intake was already correlated with BMI at T1, and BMI at T1 and T2 were also highly correlated. It seems that children with a lower BMI already may have a coping mechanism at T1 to choose healthier options after they have played an advergame promoting energy-dense snacks that triggers eating behavior, and that this is a trait that stays stable over time. Therefore, it cannot predict the difference between BMI at T1 and T2. Furthermore, we found no effect of banana intake on BMI at T2, probably because young (Dutch) children prefer apple over other fruits [32]. In other words, if children are craving for food that is induced by food advertisements, and subsequently want to choose a healthy option over the palatable snacks, they will opt more often for the fruit they prefer, in this case apple.

Furthermore, the results showed that the amount of energy-dense snacks that children consumed after playing one of the advergames or no game at all did not predict BMI at T2. Similarly, the cue-reactivity to the food cues in the advergame promoting energy-dense snacks, measured as the amount of snack food eaten after playing the game, did not predict BMI at T2. Previous studies have shown that food choices for energy-dense snacks over time do increase weight status [21, 33], but this study did not find this effect. An explanation could be that normal weight children who ate more from the energy-dense snacks compensated for this intake afterwards by reducing their normal intake, while overweight children did not. We did not measure snack intake after the experiment, but other studies have shown that food reward sensitivity and inhibitory control are important predictors of palatable food intake in adults and weight gain over time [34–36]. For example, scholars [36] have shown that adults with strong implicit preferences for snack foods and low inhibitory capacity gained the most weight over the course of one year. Another possible explanation for this null-finding is that almost 95 % of the children consumed energy-dense snacks after playing the energy-dense game, and children consumed on average approximately the same amount of energy-dense snacks within conditions, creating a small level of variation for energy-dense intake. In addition, BMI at T1 and T2 were also highly correlated. Measuring BMI after a longer period, for example 5 years, would lead to more variation in BMI, and possibly to detectable changes.

The first strength of this study is that it is the first to examine the relation between children’s susceptibility to food advertisements and their weight status two years later. Leading experts agree that food marketing is a major contributor to the worldwide childhood obesity epidemic [5], but the question still remains how food advertising exactly affects obesity. This study suggests that teaching children to consume healthier food, like apple, when impulsive reactions to food cues lead to craving for food, might help in establishing or maintaining a low BMI. Second, testing the large number of children who participated in both studies can be considered as a representative group to examine the effects of individual susceptibility to food advertisements and the development of BMI status over two years.

One limitation of this study is that we only tested food choices at one moment. If we would have tested food choices of the children with a repeated measures design, we would possibly have had a more profound insight of the association of cue-reactivity and BMI at a later stage of the children. A second limitation is that we measured BMI already after two years. As the analyses show, BMI at T1 and T2 are highly correlated, assuming that the same construct was measured. Therefore, separate analyses were conducted without and with BMI at T1 as a predictor. Measuring BMI at a later stage, for example five years later, would provide us with a more valid estimation of the relationship between food cue-reactivity after a food advertisement and future weight status.

Further research is required to unravel the lomg-term effect of reactivity to food advertisements on BMI, especially because childhood obesity remains a major health concern worldwide and food companies continue to improve their marketing strategies to effectively persuade children. The accumulation of exposure to food advertisements might be a major contributor to the obesity rates, but long-term effects of food advertising have not been examined intensively.

## Conclusion

Ths longitidunal study found a negative association among children who played an advergame promoting energy-dense snacks and subsequent apple intake on BMI two years later. The results suggest that coping with environmental cues that trigger unhealthy eating behavior is associated with the body mass index of young children at a later stage. These findings strengthen a limited evidence base suggesting that learning to respond to food cues by choosing healthy options might prevent children from excessive weight gain.

## Additional file

Additional file 1: Figure S1. Scatterplot of apple intake kcal and BMI at T1, with regression lines within conditions. **Figure S2.** Scatterplot of apple intake kcal and BMI at T2, with regression lines within conditions. **Figure S3.** Scatterplot of energy-dense intake kcal and BMI at T1, with regression lines within conditions. **Figure S4.** Scatterplot of energy-dense intake kcal and BMI at T2, with regression lines within conditions. (DOC 181 kb)

## Footnotes

1 Additional analyses were conducted with total intake or energy-dense snack intake included in the models. No significant results were found for total intake (*P* > 0.05) or energy-dense snack intake (*P* > 0.05) and the effects of the individual intake variables were not affected. Moreover, interaction effects were tested between the four conditions with the intake of jelly candy, milk-chocolate candy shells, banana, and apple, but no significant differences were found.
