# Supplementary material for: The association between BMI development among young children and (un)healthy food choices in response to food advertisements: a longitudinal study
Source: Int J Behav Nutr Phys Act. 2016 Feb 9;13:16. doi: 10.1186/s12966-016-0340-7 (PMC4748585; doi:10.1186/s12966-016-0340-7)
Supplement: Additional file 1: Figure S1. — Scatterplot of apple intake kcal and BMI at T1, with regression lines within conditions. Figure S2. Scatterplot of apple intake kcal and BMI at T2, with regression lines within conditions. Figure S3. Scatterplot of energy-dense intake kcal and BMI at T1, with regression lines within conditions. Figure S4. Scatterplot of energy-dense intake kcal and BMI at T2, with regression lines within conditions. (DOC 181 kb) [file 12966_2016_340_MOESM1_ESM.doc]

**Additional file 1**

**Additional Scatterplots**

Additional scatterplots (see Figure 1 – 4) were made to visualize the findings. As is shown in Figure 1 and 2, BMI at T1 and at T2 is strongly negatively related with apple intake among the children who played the advergame promoting energy-dense snacks, and not among the children in the other conditions. Furthermore, Table 3 and 4 show that there is no relation between energy-dense intake and BMI at T1 or at T2 for children who played the advergame promoting energy-dense snacks, or for children in the other conditions. These results imply that the children with a higher BMI did not eat less fruit because they ate more energy-dense snacks; children with a higher BMI did not eat more energy-dense snacks to satisfy the induced craving.


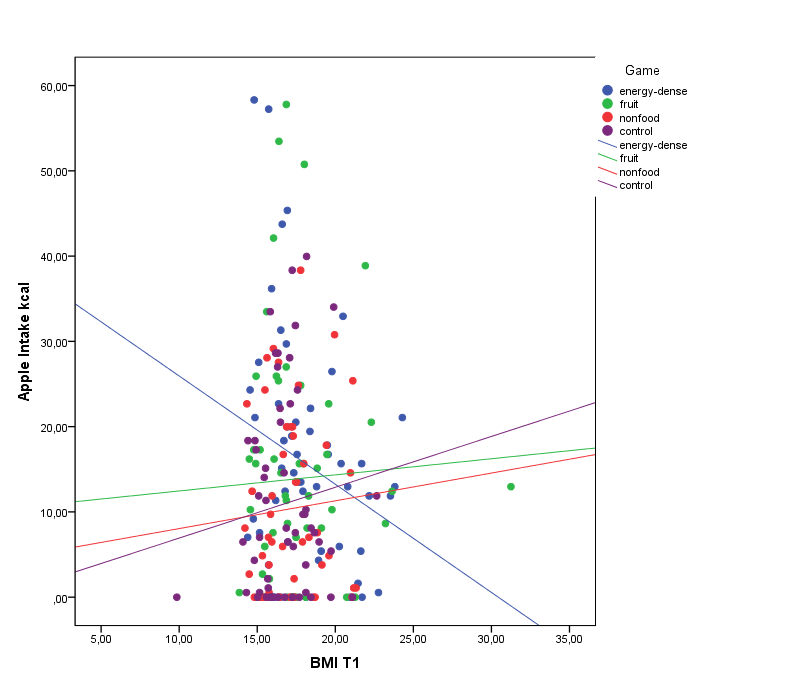


Figure S1. Scatterplot of apple intake kcal and BMI at T1, with regression lines within conditions.


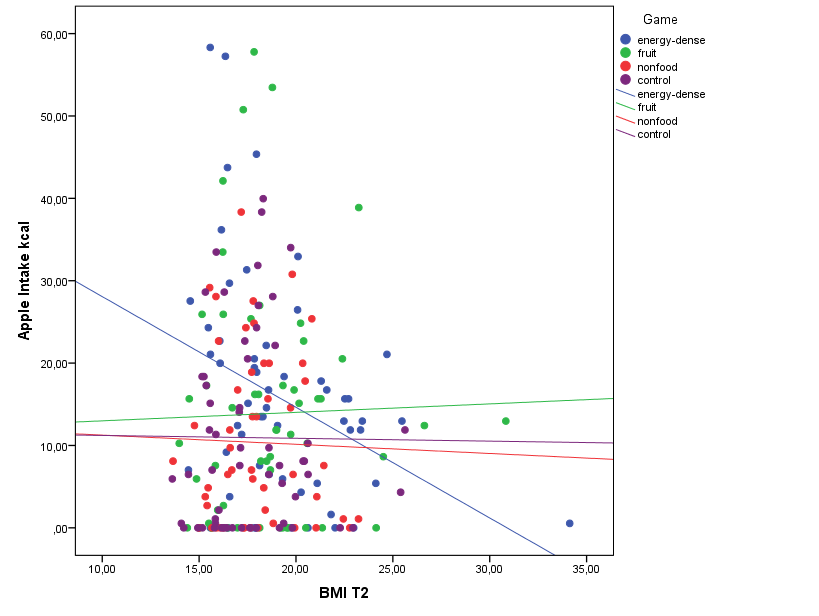


Figure S2. Scatterplot of apple intake kcal and BMI at T2, with regression lines within conditions.


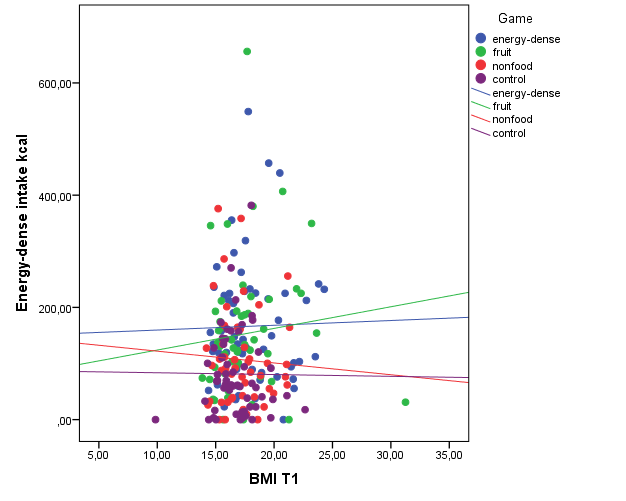
Figure S3*.* Scatterplot of energy-dense intake kcal and BMI at T1, with regression lines within conditions.


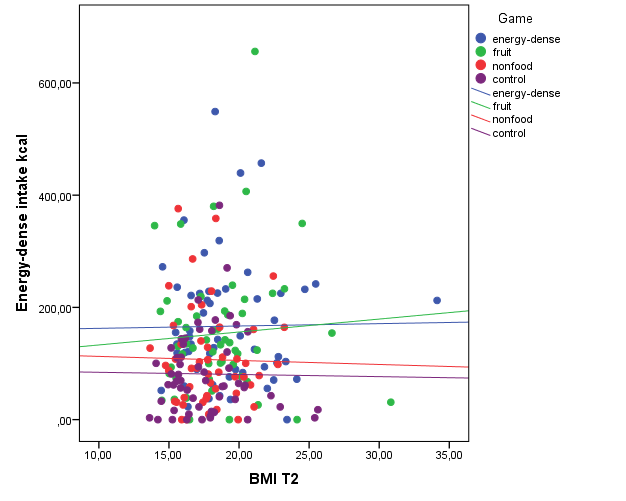


Figure S4*.* Scatterplot of energy-dense intake kcal and BMI at T2, with regression lines within conditions.
